# Editorial: Value-based nutritional intervention to reduce the progression of chronic human diseases

**DOI:** 10.3389/fnut.2023.1321289

**Published:** 2023-11-17

**Authors:** Mohammed S. Razzaque, Azeddine Atfi

**Affiliations:** ^1^Department of Pathology, Lake Erie College of Osteopathic Medicine, Erie, PA, United States; ^2^Department of Biochemistry and Molecular Biology, Massey Cancer Center, Virginia Commonwealth University, Richmond, VA, United States

**Keywords:** magnesium, phosphate, selenium, COVID-19, vitamin D

Nutritional imbalances, including in various vitamins and minerals, are associated with initiation and progression of numerous chronic disorders, including systemic and metabolic diseases (1–10). Value-based nutritional care is an approach that links the incentive for healthcare providers to the value and outcomes they deliver to patients in terms of quality, equity, and cost of care. Providing value-based nutritious foods and increasing the awareness of benefits of healthy eating habits can prevent the pathogenesis and progression of chronic human diseases. The intervention with value-based nutrition can also reduce the disease burden of patients with chronic diseases and thereby decrease overall care costs (11–15). However, before implementing the value-based nutritional intervention, the role(s) and regulation(s) of essential nutritional components in various chronic diseases need to be determined. This “Research Topic” is intended to bring together experts to share their experiences in nutritional manipulation to reduce the burden of chronic human diseases. A total of 13 articles by 82 authors have been published on this “Research Topic” to efficiently accomplish the intended objectives.

Analyzing data from the U.S. National Health and Nutrition Examination Survey (NHANES), Wang J. et al. reported that dietary magnesium intake levels and the risk of osteoporosis are negatively correlated, particularly among individuals with 55 years or older. Studies have shown that maintaining optimal magnesium and vitamin D balance improves overall skeletal functions in elderly individuals (16). In a separate study, using the same NHANES database, Wang H. et al. found that a diet with higher inflammatory potential, as measured by the Dietary Inflammatory Index, is associated with increased hyperuricemia risk, implicating dietary modification as a potential approach for hyperuricemia's prevention and control. Mironov et al. detailed the possible toxic effects of consuming a high phosphate diet for a prolonged period of time, with potential adverse effects on various organs. Phosphate-based additives and preservatives are commonly used in our daily consumed foods and drinks. Since the FDA does not mandate food industries to list phosphate content on labels, it is not easy to monitor the amount of consumed phosphate (17). Sun et al. reviewed the benefits of maintaining adequate selenium on health and disease. Selenium has claimed to have anti-oxidant, anti-tumorigenic, anti-diabetic, and immune boosting functions. The references value of selenium level varies among various populations. Moreover, selenium has a very narrow range between deficiency and toxicity. Safe modes and amounts of selenium ingestion as a supplement need to be determined. In a multi-center case-control study, conducted in the Guangdong province of southern China, a known endemic region for nasopharyngeal carcinoma, Ge et al. observed an association between levels of various serum trace elements and the risk of nasopharyngeal carcinoma. Serum levels of cadmium and manganese were positively related to nasopharyngeal carcinoma risk, which might be of clinical relevance and importance in early diagnosis of cancer patients. Li et al. proposed a prognostic significance of a novel nutritional metabolism-related scoring system (NMRS) for patients with newly diagnosed osteosarcoma. Through iterative LASSO cox analysis, an NMRS was also constructed to calculate the prognosis of osteosarcoma patients. The investigators found that NMRS can faithfully reflect patients' nutritional and metabolic status, and they predicted that by combining NMRS, patients could be further risk stratified based on existing clinical characteristics. Another skeletal lesion, spinal tuberculosis accounts for about 50% of osteoarthritis tuberculosis. Jiang et al. proposed a scoring scale to assist physicians in evaluating whether patients with thoracic and lumbar tuberculosis would develop hypoalbuminemia following surgery. The scale is simple, reliable, and has clinical guiding significance to avoid postoperative hypoalbuminemia effectively.

In a systematic review and meta-analysis, Zhang et al. elaborated on the effects of vitamin D supplementation on COVID-19 patients. They found that vitamin D supplementation reduced mortality in COVID-19 patients in cohort studies but not in randomized controlled trials (RCTs), raising an important issue of vitamin D's utility in treating COVID-19. Using umbrella meta-analysis, Musazadeh et al. detected that supplementation with L-carnitine can improve lipid profile, and they recommended L-carnitine as an adjuvant anti-hyperlipidemic agent; further clinical studies with large-scale RCTs are needed to have better therapeutic insights.

Loss of masticatory function, as a result of tooth loss is linked to the changes in food choices and inadequate nutritional intake. Using a protocol for a factorial randomized clinical trial, Qian et al. hypothesized that receiving rehabilitation of masticatory function with fixed implant dentures together with nutritional awareness is the most efficient intervention for enhancing nutrient intake in community-dwelling elderly individuals with extensive tooth loss. The results of this study have both therapeutic and policy-making implications. In a separate cross-sectional study that enrolled 3,706 participants, Qu reported a negative association between the Oxidative Balance Score (OBS) and periodontitis, suggesting that managing OBS in dietary intake and modifying lifestyle may alleviate the occurrence of periodontitis. However, such negative associations differed in the elderly and diabetes groups. Yang et al. showed that N-linoleyltyrosine ameliorates high-fat diet-induced obesity in C57BL/6 mice via regulating cannabinoid receptors, demonstrating the anti-obesity potential of N-linoleyltyrosine. As most children, who are overweight, tend to remain overweight in their adult life, causing a substantial financial burden on society. Therefore, it is crucial to target unhealthy dietary patterns in early life. Charneca et al. documented that the dietary intake of key components of a healthy diet in Portuguese preschool children is inadequate, with high intake of sugary foods and low intake of vegetables and legumes, suggesting the urgent need for nutrition education and communication strategies, including the use of technology to improve the feeding practices and develop healthy eating habits in the young children as an anti-obesity measure.

In summary, the aforementioned articles, published in this “Research Topic” highlight the nutritional aspects of various chronic diseases. These articles also identified the value of selective nutritional components and the importance of providing nutritional balance to prevent or delay the emergence of chronic diseases. Together, this “Research Topic” highlighted the need for conversation among the healthcare-providing communities to develop effective value-based nutritional strategies to promote healthier dietary habits and provide compassionate care to reduce the burden of chronic diseases and improve general health for all age groups (18).

## Author contributions

MR: Writing—original draft, Writing—review & editing. AA: Writing—original draft, Writing—review & editing.
